# Dry eye disease among Glaucoma patients on topical hypotensive medications, in a tertiary hospital, Ethiopia

**DOI:** 10.1186/s12886-021-01917-3

**Published:** 2021-03-30

**Authors:** Miraf Sahlu, Abeba T. Giorgis

**Affiliations:** Department of Ophthalmology, Menelik II Hospital, School of Medicine, College of Health Sciences, Addis Ababa University, P.O.Box 8079, Addis Ababa, Ethiopia

**Keywords:** Dry eye disease, Glaucoma, Hypotensive medication

## Abstract

**Background:**

Dry eye disease is a multifactorial disease; causing various ocular symptoms with potential damage to the ocular surface. Applying hypotensive eye drops are presumed to initiate or exacerbate existing dry eye disease. The purpose of this study was to determine the frequency of signs and symptoms and severity of dry eye disease among glaucoma patients on topical hypotensive medications and controls.

**Methods:**

A cross-sectional comparative study, involving 320 glaucoma patients and controls. Ocular Surface Disease Index (OSDI) symptoms score and Schirmer, tear breakup time and corneal staining tests were used to assess dry eye disease. Data was analyzed using SPSS version 24 software; *p*-value less than 0.05 was considered as statistically significant.

**Results:**

Among the 160 study glaucoma patients, the mean duration of topical hypotensive medication use was 5.2 ± 5.21 years (range, 4 months - 32 years). Mild to severe level of OSDI score was found in 122 (76%) glaucoma patients and in 137 (86%) controls (*p* = 0.033). Mild to sever abnormal clinical tests in the glaucoma patients and control, respectively, were 106 (66%) vs 80 (50%) corneal staining (*p* = 0.045), 79 (49%) vs 72 (45%) TBUT (*p* = 0.021), and 91 (57%) vs 83 (52%) Schirmer test (*p* = 0.242). Test results at the level of sever: 2 (1%) vs 0 (0%) corneal staining, 50 (31%) vs 39 (24%) TBUT and 65 (41%) vs 60 (38%) Schirmer test in the glaucoma patents and controls, respectively. Corneal staining and TBUT had correlation with the number of drugs (*p* = 0.004 and 0.031, respectively), and more relationship of the two tests with total number of drops applied per day (*p* = 0.01 and *p* <  0.001, respectively). Patients on pilocarpine and timolol had more corneal staining and lower TBUT [(*p* = 0.011 and *p* <  0.001) and (*p* = 0.04 and 0.012), respectively].

**Conclusions:**

The study has identified glaucoma patients to be more affected by dry eye disease than non-glaucoma patients, and presence of significantly lower TBUT and higher corneal staining in the glaucoma patients on multidrops and multidose per day. We recommend consideration of evaluation and management of DED for glaucoma patients on multidrops and multidose hypotensive medications.

## Study design

Impact of hypotensive drops on dry eye disease: A cross-sectional comparative study involving glaucoma patients on hypotensive drops and control.

## Background

Dry eye disease (DED) or ocular surface disease (OSD) is a multifactorial disease of the eye. The disease is characterized by failure to produce high quality or sufficient amounts of tears to moisturize the ocular surface. The disease causes symptoms of discomfort, visual disturbance and tear film instability with potential damage to the ocular surface [1]. The prevalence of DED ranges from 5 to 50% globally and 6.8% in USA [2, 3]. It is more common among older age people and in women than men (8.8% vs 4.5%) [3].

Among the risk factors for DED are ocular and systemic medications [4]. Glaucoma, which mainly affects people age 40 and above, is commonly treated with either single or multiple hypotensive eye drops [5]. It is known that applying hypotensive eye drops over long period of time to either initiate or exacerbate existing dry eye disease. Therefore, glaucoma patients have double risk to be affected by DED [6–10].

Studies have been using structured scoring format including ocular surface disease index (OSDI), standardized patient evaluation of eye dryness (SPEED), and dry eye questionnaire (DEQ-5) to quantify the severity of DED/OSD symptoms [11–13]. Clinical tests that have been used to assess the tear volume, and the extent of ocular surface epitheliopathy are Schirmer test and ocular surface staining using fluorescein, rose Bengal and lissamine green, respectively [14].

The diagnosis of DED in glaucoma patients is often overlooked as the focus of management is on controlling the eye pressure; even if, DED has impact on drug adherence and the quality of life [15]. Therefore, the purpose of this study was to assess the frequency of signs and symptoms and severity of dry eye disease among glaucoma patients on topical hypotensive medications and controls.

## Methods

A hospital-based cross-sectional comparative study was conducted at a glaucoma unit and general out patient clinics of the department of ophthalmology, Menelik II tertiary referral hospital, Ethiopia from October 1, 2019 to November 15, 2019. The source populations for this study were all glaucoma patients who received services at the glaucoma unit and ophthalmic patients attending the general out patient clinics. All glaucoma patients who had follow-up visit and new ophthalmic patients (controls) who came for care during the study period were the study population.

### Sample size determination and sampling technique

The sample size was calculated using the formula for comparative studies [16]. Taking the proportion from other similar studies [17], it was assumed that the minimum prevalence of DED among glaucoma patients to be 39% and that of non-glaucoma patients 25%. The significant level was taken as 95% with alpha error set to 5% and power of the study was set at 80%. The ratio of the cases to the control was taken as one. Using these parameters, the required sample size came to be 160 for each group.

### Recruitment of study participates

All glaucoma participants were recruited consecutively using their charts during regular scheduled glaucoma unit appointment days. The controls were new patients attending the general ophthalmic outpatient clinics of the department; similarly, they were recruited consecutively using their charts from a triage room**.** Ophthalmic/general nurses measured both visual acuity and intraocular pressure, and document on the patient’s chart prior getting eye care service form physicians. While the patients were waiting in the waiting area, they were screened for eligibility based on the inclusion and exclusion criteria using their charts, history and examination under slit lump microscope. The selected patients were briefed about the purpose of the study, the questionnaires and the clinical tests, and then requested for willingness to participate. Eye that fulfilled the inclusion criteria was selected, and when both eyes met the inclusion criteria, the right eye was taken.

### Inclusion and exclusion criteria

Inclusion criteria for the glaucoma patients were: 1) age above 40, 2) diagnosed with any type of glaucoma and 3) on topical hypotensive medication (s) for three months and above. Exclusion criteria were: 1) use of any non-hypotensive topical drops within the previous 3 months, 2) active or recent ocular infection, 3) presence of immune-compromising disease, 4) lid abnormality such as ectropion, entropion, lagophthalmous, trichiasis and blepharitis, 5) prior lid or ocular surgery and 6) any ocular surface lesions. The controls were also age 40 and above and the exclusion criteria were the same as for the glaucoma patients.

### Data collection and analysis

The participants were given OSDI questionnaire to be filled by themselves or by assistant (accompany person or ophthalmic nurse) if they couldn’t do it. The OSDI questionnaire (Allergan Inc., Irvine, CA), which is originally written in English was translated into a national language, Amharic, for the purpose of this study, and was verbally translated for those who speak other languages by accompany persons or others.

When answering the questionnaires if the participant didn’t do any of the activities in the questionnaire, for example doesn’t watch TV, he / she didn’t have to answer the specific question related to watching TV. The score was calculated out of the total questions answered by the participants not by all the questions asked. Each question of OSDI is graded from 0 to 4: 0 indicating none of the time, 1 some of the time 2 half of the time, 3 most of the time, and 4 all of the time.

The participants were also interviewed about their sociodemographic background. For the glaucoma patients, information on hypotensive drops including duration of use, type, number and frequency of drops application was obtained using an interviewer administered structured questionnaire. Patients’ charts were used to retrieve information on the type of medication(s) if patients were not sure during the interview, and the diagnosis and stage of glaucoma. Then patients of both groups under went through the three standard clinical tests. The tests were performed in the following order.

#### Schirmer I test (without anesthesia)

Each participant was asked to look up and the lower eyelid was drawn gently downward on the temporal side. Rounded bent end of a sterile Schirmer strip (Iscon Surgical Ltd., An ISO 9001, Marudhar, India) was hooked in the inferior fornix over the junction of the temporal and central one third of the lower eyelid margin with care taken to avoid touching the cornea; then the patient was asked to close his/her eyes. After 5 min, the strip of filter paper was removed and the amount of wet filter paper was measured and recorded in millimeters. If the tear front moved unevenly, it was measured from the notch to the middle of the diagonal line. Only whole numbers rounded up to the next whole number were recorded if the tear front is at or greater than the half-millimeter mark.

#### Tear break up time (TBUT)

One drop of topical anesthesia - tetracaine, was instilled and the patient was asked to close his/ her eyes. After 1 min the patient was instructed to look up to apply a fluorescein sodium ophthalmic strip into the inferior fornix, blink 5 times and then to hold the eyes open. The cornea was scanned with slit lamp microscope using a cobalt blue filter at 10 X magnification. A dry area was indicated by the appearance of a black spot or line. The time in seconds between the last blink and the appearance of black spot was recorded using stopwatch as a tear film break-up-time.

#### Corneal and conjunctival fluorescein staining

Under slit-lamp microscope with 10X magnification, extent of staining of the conjunctiva and cornea was assessed and graded against the standard Oxford chart [18].

Operational definitions: OSDI questionnaire was graded as normal 0–12, mild to moderate 13–32 and severe 33–100. The value of OSDI was calculated as sum of the severity for all questions answered divided by 4X total number of questions answered.

Schirmer test: normal > = 10 mm, mild to moderate 6-9 mm and severe 0-5 mm. The TBUT graded as normal > = 10 s, mild to moderate 5–9 s and severe < 5 s. Corneal fluorescein staining: normal if 0 (no staining), mild I or II, moderate III and Severe IV or V. Non normal grades were considered as abnormal results.

Data quality control and analysis: At the end of each day, all of the collected data was reviewed and checked for completeness and entered into SPSS (Statistical Package for the Social Science; SPSS Inc., Chicago, IL) version 24 software for data analysis. Descriptive statistics such as frequencies, percentages and mean were used to summarize the demographic data, drug duration, type of medications and OSDI score and results of the three clinical tests. Pearson’s correlation, t-test and one-way ANOVA were utilized for further analysis when indicated. *P*-value < 0.05 was considered to be statistically significant.

#### Ethical consideration

The study was carried out in accordance with the tenets of the Declaration of Helsinki, and got ethical approval from the research and publication ethics committee of the Department of Ophthalmology, College of Health Sciences, Addis Ababa University. Written informed consent was obtained from each participant involved in the study. Patients with tests indicating dry eye were informed; the results were documented on their chart to be seen and managed by the treating physicians.

## Result

A total of 320 patients involved in the study, 160 glaucoma and 160 control patients. The details of sociodemographic characteristics of both groups are summarized in Table 1. The mean ± SD age was 62.2 ± 11.58 (range, 41–87) years in the glaucoma patients and 61.4 ± 10.76 (range, 40–90) years in the controls. Male to female ratio was similar in both groups (1.1:1 and 0.9: 1, respectively).

**Table 1:** Sociodemographic Characteristics of the Glaucoma Patients and Controls

Characteristic	Glaucoma Patients No (%)	Controls No (%)	***P*** Value*
**Age (years)**			
40–50	32 (20)	28 (18)	
51–60	40 (25)	48 (30)	
61–70	53 (33)	59 (37)	0.51
71–80	27 (17)	19 (12)	
> 80	8 (5)	6 (4)	
**Sex**			
Male	85 (53)	76 (48)	0.31
Female	75 (47)	84 (53)	
**Address**			
Urban	112 (74)	136 (85)	0.18
Rural	48 (21)	24 (15)	
**Occupation**			
Indoor	112 (70)	127 (80)	0.055
Outdoor	48 (30)	33 (21)	
**Educational status**			
Illiterate	45 (28)	23 (14)	
Primary	55 (34)	29 (18)	< 0.001
Secondary	24 (15)	47 (29)	
College & above	36 (23)	61 (38)	

*Paired sample t-test was used to compare the categorical variables

Primary open angle glaucoma was the commonest diagnosis (87, 54%) followed by Pseudoexfoliative glaucoma (65, 41%). The glaucoma patients had been using 7 types of hypotensive eye drops; none of which were preservative free. The types of drops were timolol (53%), timolol and dorzolamide fixed-combination (41%), pilocarpine (36%), latanaprost (12%), brimonidine (25%), betaxolol (2%) and dorzolamide (1%). The mean ± SD duration of topical medication use was 5.2 ± 5.21 years (range, 4 months - 32 years). The minimum and maximum frequency of drop(s) per day was 1 and 6. Two and 5 drops per day were the common frequent regimes (51 and 34%, respectively).

The mean ± SD of DED symptom with OSDI score was 30.8 SD ± 22.9 (range, 0–100) in the glaucoma patients, and 36.1 ± 21.3 (range, 0–86) in the control, *p* = 0.033. Mild to severe level of OSDI value was detected in 122 (76%) glaucoma patients and 137 (86%) controls. The level was severe in 69 (43%) glaucoma patients and 85 (53%) controls, *p* = 0.028.

Table 2 shows the results of the 3 clinical tests among the glaucoma and control patients; a paired sample t-test comparing the mean values of the two groups for each test. The difference between the glaucoma and control participants with regard to corneal staining and TBUT was statistically significant. Abnormal (mild – sever) corneal staining was detected in 106 (66%) glaucoma patients and 80 (50%) controls, *p* = 0.045. Tear breakup time was abnormal in 79 (49%) glaucoma patients and 72 (45%) controls, *p* = 0.021. While Schirmer test was abnormal in 91 (57%) glaucoma patients and 83 (52%) controls, *p* = 0.242. The glaucoma patients had higher number with 2 or 3 abnormal clinical test results (98 vs 77), while the controls had more normal results in all the 3 clinical tests than the glaucoma patients (39 vs 21).

**Table 2:** Clinical Tests of Dry Eye Disease of Glaucoma Patients and Controls

Test	Glaucoma Patients		Controls		P Value*
	No	%	No	%	
**Corneal Staining**					
Normal	54	33	80	50	**0.045**
Mild	90	56	66	41	
Moderate	14	9	14	9	
Severe	2	1	0		
**TBUT**					
Normal	81	51	88	55	0.021
Mild to moderate	29	18	33	21	
Severe	50	31	39	24	
**Schirmer**					
Normal	69	43	77	48	0.242
Mild to moderate	26	16	23	14	
Severe	65	41	60	38	
**Total number of abnormal tests**					
Zero/all normal	21	13	39	25	0.034
One	41	26	44	28	
Two	59	37	40	25	
Three	39	24	37	23	

* t-test was used for mean difference; TBUT = Tear Break up Time

**Fig. 1:**
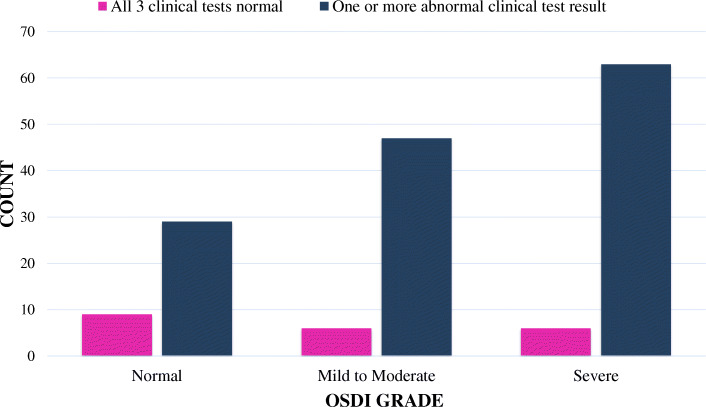
Relationship between OSD symptoms & clinical test results in glaucoma patients

Pearson’s correlation coefficient was used to see any correlation between the OSDI results and the 3 clinical tests. For the glaucoma patients, the OSDI results had statistically significant correlation with the overall number of abnormal tests (*p* = 0.036) (Fig. 1), and a strong correlation with Schirmer test (*p* = 0.009). On the other hand, the correlation of OSDI score with corneal staining and TBUT was not significant (*p* = 0.149 and 0.126 respectively).

The results of OSDI questionnaire and the clinical tests were dichotomized into normal versus abnormal as seen in Table 3 for the glaucoma Patients. The analysis showed that those who had abnormal test results were 3 times more likely to report DED symptoms than those with normal results in all the three clinical tests (odd ratio = 3.00 with 95% CI 1.5–7.9, *p* = 0.022). For the control, the OSDI results didn’t correlate significantly with the number of abnormal tests (*p* = 0.201).

**Table 3:** Agreement between OSD symptoms and clinical test results in glaucoma patients

		TBUT		SCHIRMER		CORNEAL STAINING	
		Normal	Abnormal	Normal	Abnormal	Normal	Abnormal
OSDI	Normal	25	14	21	18	12	27
	Abnormal	56	65	47	74	43	78
OR: 95% CI *P* value		2.0 (0.98–4.3) 0.039		1.84 (0.88–3.8) 0.10		0.8 (0.37–1.7) 0.04	

One-way ANOVA analysis was utilized to find out any difference among the different sociodemographic characteristics and OSDI questionnaires score and the clinical test results in both the glaucoma patients and controls, which showed no statistically significant difference. There was no significant difference among the different educational levels with the result of the clinical tests and the OSDI score; except the TBUT result of the glaucoma patients which had mean value of 7.6 ± 4.6 s vs 11 ± 6.0 s (*p* = 0.03) in those who can’t read and write versus those with college and above educational level, respectively.

To see the effect of polypharmacy on abnormal test results, the glaucoma patients were divided and analyzed as those applying only one type of hypotensive medication and those applying 2 or more medications. The analysis showed a statistically significant mean difference in total number of abnormal tests between the two groups, 1.5 + 0.96 vs 1.9 + 0.96, *p* = 0.013.

The mean + SD number of drops applied per day in early, moderate and advanced stage of glaucoma were 2.5 + 1.14, 3.0 + 1.37, 3.7 + 1.46, respectively. One-way ANOVA analysis revealed the presence of higher mean corneal staining in the advanced stage as compared to the early and moderate stage of glaucoma, 1.4 + 1.13 vs 0.6 + 0.66 (*p* < 0.001) and 1.4 + 1.13 vs 1.0 + 0.90, *p* = 0.047, respectively. Additionally, TBUT was lower (7.6 + 5.1 vs 10.7 + 4.97 s, *p* = 0.009) and the number of abnormal tests was higher (1.9 + 0.94 vs 1.4 + 0.97, *p* = 0.014) in advanced stage than early and moderate stage of glaucoma. But the stage of glaucoma didn’t correlate significantly with OSDI score and the Schirmer test. There was no significant difference between the types of glaucoma (POAG, PXG, OHT) and the OSDI score and the number of abnormal tests.

**Fig. 2:**
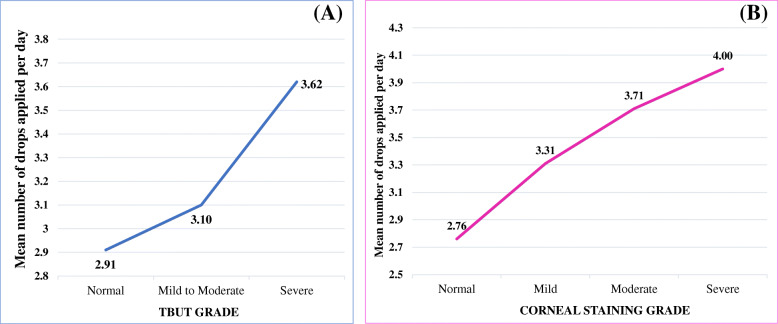
Relationship between mean number of drops applied per day with Tear Break Up Time (**a**) & corneal staining (**b**)

Pearson’s correlation was also used to see if there was a relationship of the number of hypotensive drugs, total number of drops applied per day and drug duration in years with the 3 clinical tests and the OSDI score. The corneal staining and TBUT had significant correlation with the number of drugs (*p* = 0.004 and 0.031, respectively); even more significant relationship of the two tests with total number of drops applied per day (*p* = 0.01 and *p* < 0.001, respectively). Duration of drop application had no significant association with corneal staining and TBUT. Figure 2 shows the relationship between the grades of TBUT and corneal staining with the number of drops applied per day. Schirmer test and OSDI score had no statistically significant correlation with the number of drugs, drops per day or duration of medication use.

T-test was used to compare those applying one type of drug to those on other drops in relation to the OSDI and the 3 clinical tests. Those on pilocarpine had statistically significant lower TBUT and more corneal staining results than those not applying the drug (*p* = 0.011 and *p* < 0.001, respectively). Besides, those on timolol or combination of timolol and dorzolamide had statistically significant abnormal test results than those not applying the drug (*p* = 0.04 and *p* = 0.012 respectively).

## Discussion

This study assessed dry eye disease in glaucoma patients on topical hypotensive drops and controls using OSDI symptoms score and three clinical tests (corneal staining, tear breakup time and Schirmer test).

The similarity in mean age, 62.2 ± 11.58 and 61.4 ± 10.76 years, and female sex proportion, 75 (47%) and 84 (53%), in the glaucoma patients and controls, respectively, could help to balance the age and sex related ocular surface changes which are the leading risk factors for dry eye disease [1–3].

The mean ± SD of DED symptom with OSDI score was higher in the controls than the glaucoma patients (36.1 ± 21.3 vs 30.8 ± 22.91). In addition, both mild to severe and severe level of OSDI were higher in the controls (137, 86% and 85, 53%) than in the glaucoma patients (69, 43% and 69, 43%). The controls were new patients who had not received any ophthalmic attention at the hospital; thus, their response to the OSDI questionnaire might be exaggerated to get attention than the glaucoma patients who had been already diagnosed, on treatment and follow-up. On the other hand, the glaucoma patients might be more concerned about their glaucoma than other symptoms. Besides, the OSDI score of the controls has poor correlation with the clinical test results. Such poor correlation was also found in other studies and the potential explanation given were reporting bias by patients, heterogeneity of the disease and at times paucity of symptoms in a subset of patients with severe disease [19, 20]. On the other hand Barisic et al. has reported mild to sever OSDI in 75% of 110 glaucoma patients on topical hypotensive medications and in 30% of 50 controls [21].

Comparing to other study reports on glaucoma patients, our participants in both groups had higher percentage of abnormal OSDI results (76% in the glaucoma and 86% in the controls), and reported more severe symptoms, 43% glaucoma patients and 53% controls [8, 9].

As depicted in Table 2, the glaucoma patients had more abnormal results in the three clinical tests than the controls (corneal staining 66% vs 46%, *p* = 0.045, TBUT 49% vs 45%, *p* = 0.021 and Schirmer 57% vs 52%, *p* = 0.242). These results are in line with a similar cross-sectional comparative study by Ramli et al. that reported higher percentage of abnormal tests in the glaucoma group than the control (corneal staining 63% vs 36%, *p* = 0.004 and Schirmer 39% vs 25%, *p* = 0.049) [17].

The glaucoma patients were on 7 different hypotensive eye drops with benzalkonium chloride (BAC) preservative, which are additional risk factors for DED [6, 7, 22]. The higher ocular surface disease signs in the glaucoma patients than the controls can be explained by the toxic effect of BAC preservative and active molecular effect of the medication(s) on the corneal epithelial cells [23]. Moreover, Cha and his colleagues have proven BAC to induce corneal epithelial lysis and even more dysfunction with higher concentration on their experimental study on cultured rabbit corneal epithelial cells [24].

Considering the severity of the clinical tests in this study, the proportion of severe results in the glaucoma patients and the controls were Schirmer test 41% vs 38%, TBUT 31% vs 24% and corneal staining 1% vs 0.0%. There is difference and similarity when these severity results are compared with a cross sectional study on the prevalence of ocular surface disease in 101 glaucoma patients by Leung et al. that reported the presence of severe tear deficiency: Schirmer test in 35%, TBUT in 65% and corneal staining in 0% [8]. The figure variations could be due to the involvement of both eyes and taking the result in at least one eye, while in our study the involvement was only one eye of each patient; still the data are indicative of the extent of DED in glaucoma patients. Trachoma, vitamin A deficiency and higher environmental temperature are the additional risk factors for DED that has to be considered among Africans [25]. This can be considered as one explanation for the higher test results in our study participants.

In this study, even though, OSDI symptom score was higher in the controls, its correlation with the overall number of abnormal clinical tests was significant (*p* = 0.036) in the glaucoma patients. Besides, those who had abnormal test results were 3 times likely to report symptoms (odd ratio = 3.00 with 95% CI 1.5–7.9, *p* = 0.022), which was not the case for the controls (Table 3). This indicates the relevance of testing to confirm the presence of DED in symptomatic patients, and also asking glaucoma patients on topical drops if they have symptoms in order to recognize and treat the disease that has impact on patient quality of life and medication adherence [7]. Considering the age and eye medications related risks for DED, it is reasonable to consider DED evaluation as part of glaucoma patient management.

Depending on the stage of glaucoma, patients with advanced stage were found to apply more number of medications (3.7 + 1.46 SD) than those with early and moderate stage of glaucoma. In relation to this, the analysis showed the presence of higher corneal staining, lower TBUT and higher abnormal tests in the advanced stage. Moreover, both corneal staining and TBUT were found to have correlation with increased number of drugs (*p* = 0.004 and 0.031, respectively) and even more significant correlation with total number of drops applied per day (*p* = 0.01 and *p* < 0.001, respectively) (Fig. 2). These finding can be explained by the likelihood of increased adverse effects of the medications and/ or their preservative (BAC) on the ocular surface and the quality of tear film, which could be dose and drug number dependent [15, 26].

The analysis finding of the presence of association of lower TBUT and more corneal staining with pilocarpine use and drops containing timolo in this study can be supported by an experimental study on the influence of pilocarpine and timolol on human meibomian gland epithelial cells by Zhang et al. that detected the presence of direct effect of these drugs on the morphology, survival and proliferation capacity of the gland cells [27]. Moreover, pilocarpine’s tear film pH lowering effect has been reported by Azuamah et al. [28]. Therefore, based on our findings and those study reports, we may consider direct effect of these drugs on the ocular surface in addition to their preservative.

Limitations of the study: All the participants were using drops with BAC as a preservative and the effect of preservative-free drugs was not seen in this study.

## Conclusions

The study has identified glaucoma patients to be more affected by dry eye disease than non-glaucoma patients, and the presence of significantly lower TBUT and higher corneal staining in the glaucoma patients on multidrops and multidose per day. We recommend consideration of evaluation and management of DED for glaucoma patients on multidrops and multidose hypotensive medications.

## Data Availability

The datasets used and analyzed during the current study are available from the first author on reasonable request.

## Abbreviations

DED: Dry eye disease
OSDI: Ocular Surface Disease Index
TBUT: Tear break Up Time
SPSS: Statistical Package for the Social Science
PXG: Primary open angle glaucoma (POAG), Pseudoexfoliative glaucoma
OHT: Ocular hypertension
